# Sir Peter Eade

**Published:** 1916-09-09

**Authors:** 


### SIR PETER EADE.

Sir Peter Eade, who died last year at the ripe age of ninety, has left behind him a very brief autobiography and a very bald diary, which have been edited by Dr. Sydney Long, and are now published. Frankly, the volume is disappointing. A lively and detailed record of life as it was lived in Peter Eade's early days would have been of very great interest. He lived when rushlights were still used in the bedroom and the kitchen, before the flint and steel and tinder had been superseded by the lucifer match; he had seen a man sitting in the stocks for punishment, he had travelled by coach before the days of railways; and, as to medical practice, he saw operations before the employment of anæsthetics; he witnessed an epidemic of diphtheria when it was a new and undescribed disease; he was himself the first to draw attention to the paralysis which sometimes follows it; he made for his own or his father's practice galenical preparations from herbs grown for the purpose in his own garden; and yet of all these things he tells us almost nothing, and of many another extinct custom that he must have witnessed he tells us nothing at all. His diary is equally disappointing. He was for many years a very prominent figure in Norwich, the capital of East Anglia; he served as Sheriff for his city, and three times as its Mayor; he took a large share in every scheme and project set on foot for the benefit of the city, and himself initiated many of them; he met, either officially or privately, many of the most celebrated men of his time; and yet his diary is a bare record of events without detail and without comment. He tells us nothing of the difficulties that he surmounted, nothing of the adroit diplomacy and tenacious persistence with which he surmounted them. He tells us nothing of the conversations he had with celebrated people, nothing of his meetings with the then Prince of Wales, with Gladstone, Harcourt, Huxley, and many another prominent Victorian. He tells us that he went down the River Yare to Yardley Cross to read three times the quaint proclamation of the rights of the Norwich Corporation over the river, but he tells us nothing of the terms of the proclamation, nor how, in this desert spot far from human habitation, it calls upon objectors to come forward and declare their grievances against the management of "the King's Majesty's river of Wensum." His reticence is the more remarkable that he was not without literary tastes or literary ability. He not only wrote on medical subjects, but made contributions to pure literature. He was the author of a history of the parish of St. Giles, in which he lived for so many years, of a history of the Norfolk and Norwich Hospital, and other publications.

Though we must regret that his autobiography is not more interesting, yet we cannot but admire the life that it records, a life in which every minute seems to have been occupied with busy industry animated by public spirit and benevolence. There was scarcely an institution in Norwich of which Sir Peter Eade was not, or had not been, either president, or chairman, or director, or in some position of authority and influence, nor one whose affairs he did not make it his business to investigate nor whose interest and welfare he did not further. He was a conspicuous example of the class of men so numerous in this country, whose great business aptitude is accompanied by great public spirit, who give freely and ungrudgingly of their time, thought, labour, and money to their fellow-citizens and to public affairs, and who contribute so signally to the strength and the welfare of their country.

